# Supplementary material for: Optical experiment to test negative probability in context of quantum-measurement selection
Source: Sci Rep. 2019 Dec 13;9:19021. doi: 10.1038/s41598-019-53121-5 (PMC6910950; doi:10.1038/s41598-019-53121-5)
Supplement: Supplementary file 1 — Supplementary Information [file 41598_2019_53121_MOESM1_ESM.pdf]

# Supplementary Information: Optical experiment to test negative probability in context of quantum-measurement selection

Junghee Ryu,[1,*] Sunghyuk Hong,[2] Joong-Sung Lee,[2] Kang Hee Seol,[2] Jeongwoo Jae,[2] James Lim,[3] Jiwon Lee,[2] Kwang-Geol Lee,[2,†] and Jinhyoung Lee[2,‡]

[1]*Centre for Quantum Technologies, National University of Singapore, 3 Science Drive 2, 117543 Singapore, Singapore*
[2]*Department of Physics, Hanyang University, Seoul 04763, Republic of Korea*
[3]*Institute of Theoretical Physics and Integrated Quantum Science and Technology IQST, University of Ulm, Albert-Einstein-Allee 11, D-89069 Ulm, Germany*

## I. ERROR ANALYSIS

Experimental errors occur when optical devices (components) have imperfect alignment and/or response. We characterized the optical components used in our measurements to obtain their operational errors; PBSs, waveplates, avalanche photodiodes (APDs, Perkin Elmer, SPCM AQ4C). For example, the angles of the wave-plate are controlled by motorized rotational stages, and the operating error is less than 0.5 degree. This results in approximately 1 degree of maximum error for the input polarization, which leads to 1.1% error in the detected counts at the APDs. Also, the extinction ratio of the PBS (CVI Laser Optics, PBS-800-050) exceeds $10^3$. This is true in the transmitted part ($< 0.1\%$ error), but the reflected beam contains about 5% of incorrectly polarized photons. The four APDs are corrected using the reference detection efficiency as described in the detection scheme in the Method section. However, this cannot be perfectly accomplished. Thus, we consider the 5% error including the fluctuation of the APD's working efficiency. The total error in the measurement of a quantity is calculated as $\sum_i \sqrt{(\text{error of each component}_i)^2 \times (\text{times used})}$. Here, we assumed that there is no correlation between the errors of different components. Note that the given error values (error bars in the figures) are maximally estimated. The statistical fluctuation over measurements at many time intervals can be inferred from the distribution of the experimental values (red circles) in Fig. 2a in the main text, which is much smaller than the given error bar.

## II. SECOND ORDER CORRELATION FUNCTION OF THE RESOURCES

We examined the anti-bunching characteristics of the heralded single photons and single photons from a single molecule by measuring the second-order correlation function $g^{(2)}(\tau)$. In the case of the SPDC source, as shown in Fig. S1a, photon pair is initially separated using a PBS into the signal and the idler paths. Both paths are then sub-divided by the 50:50 non-polarizing beam splitter (NPBS) into two branches. By combining one branch of the signal and one of the idler using an AND gate, it becomes possible to mimic the coincidence of the heralded photon. Therefore, the Hanbury-Brown and Twiss (HBT) measurement of the outputs of two AND gates implies the $g^{(2)}(\tau)$ of the heralded photons. Two digital chips (PO74G08) are used for the AND gates. The HBT measurement was performed using the start-stop mode of a TCSPC device (PicoQuant, PicoHarp 300). The resulting experimental curve is shown together with a theoretical calculation in Fig. S1b. In the calculation, we considered the following system parameters; the timing jitter of the APDs (= 0.61 ns) and the coincidence time window of the AND gates (= 5.5 ns). The average value of the dip in the time delay range of -3 ns $\sim$ 3 ns is only 0.036. The near zero value of $g^{(2)}(0)$ ensures that the heralded photons are the most similar to the single photons. For the case of the single molecule, the emitted photons are divided by a NPBS into two branches and are directly used as the inputs of the start-stop measurements. The results are shown in Fig. S1c.

## III. ADDITIONAL EXPERIMENT BY POST-SELECTED WEAK FIELD

We here discuss the negativity of the operational quasiprobability using a weak-field. Given that such light does not exhibit the anti-bunching characteristic, the weak-field can be regarded as classical light. However, we can detect the negativity with a post-selection process. This indicates that our method provides an operational way to detect the nonclassicality of optical fields within the context of selecting measurement procedures.

The input source was prepared as follows; we used picosecond pulses from a mode-locked Ti:sapphire laser (Mira 900). The centre wavelength is set to 800 nm and the pulse repetition rate is reduced down to 3.8 MHz with a pulse picker (Coherent 9200). Then, using neutral density filters, the intensity of the beam is attenuated so that the average number of photons range from $10^{-3}$ to $10^{-1}$ per pulse. We implemented the same measurement setups as shown in Fig. 1 in the main text. We post-selected the raw data to evaluate the negativity in a way that only single APD clicks were sampled and the rest

* rjhui82@gmail.com
† kglee@hanyang.ac.kr
‡ hyoung@hanyang.ac.kr

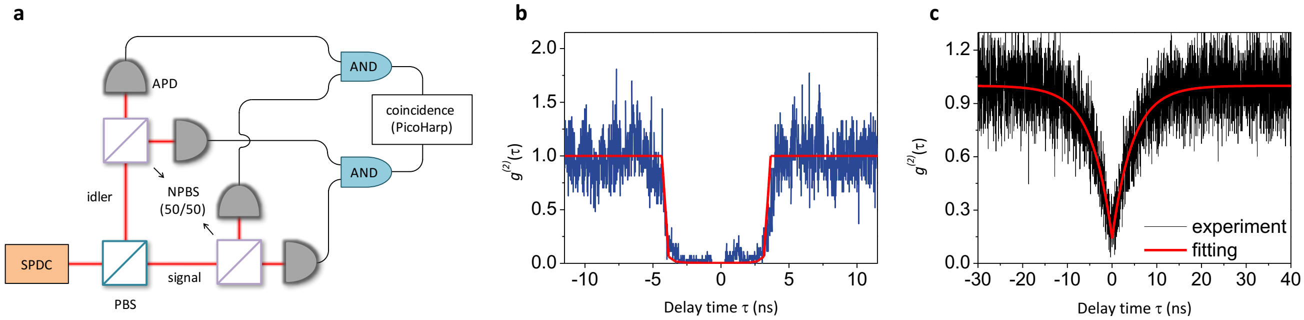


FIG. S1. **Second order correlation function of the resources.** **a**, Experimental schematic for measuring the anti-bunching property of heralded single photons [1, 2]. **b**, Experimental (blue line) and theoretical values (red line) of the second order correlation function of heralded single photons. Average value of $g^{(2)}$ dip is 0.036. **c**, The $g^{(2)}(\tau)$ function of single photons emitted from a single terrylene molecule. The result reads $g^{(2)}(0) = 0.14$.

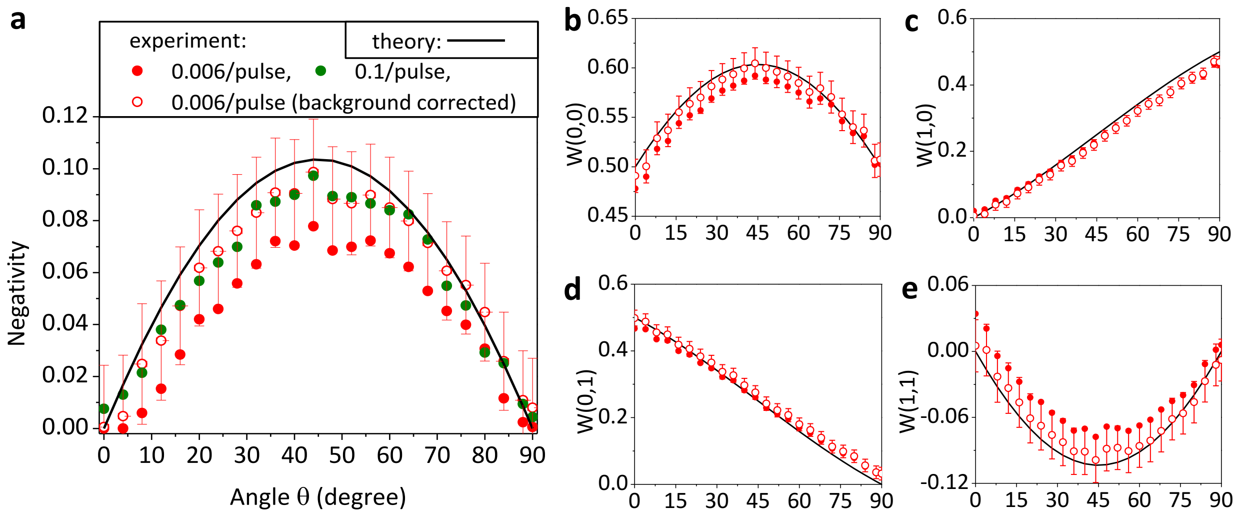


FIG. S2. **Negativity for a weak field input.** **a**, Negativity for two different input intensities (average photon number): Green circle for $1 \times 10^{-1}$ per pulse, and red circle for $6 \times 10^{-3}$ per pulse. When the dark-count of the APDs is corrected, the experimental results are in closer agreement with the theoretical data as shown by the red open circles. The error bars are shown only for the red open circles for clarity. Each component of the quasiprobability distribution $\mathcal{W}(a_1, a_2)$ is presented in **b-e** as a function of the angle $\theta$ with a fixed $\phi = 0°$ of the state $|\Psi(\theta, \phi)\rangle = \cos(\theta/2)\,|H\rangle + e^{i\phi}\sin(\theta/2)\,|V\rangle$.

of events, e.g., more than two clicks simultaneously were neglected.

Experimental results together with the theoretical predictions are presented in Fig. S2a for the negativity and in Fig. S2b-e for each $\mathcal{W}(a_1, a_2)$. We started with the average photon number $6 \times 10^{-3}$ per pulse (red open and filled circles in Fig. S2a). The maximum negativity was obtained as 0.078 without the correction of the dark count of the APDs (red filled circle). This maximum increased to 0.099 after correcting for the dark count (red open circles); we measured the dark count of each APD and subtracted this value from the total measured counts. For a higher average photon number ($10^{-1}$ per pulse), the maximum negativity was 0.097 even without the correction for dark-count (see green circle). This is because for a higher detection count, the contribution of the dark count of the APD ($\sim 10^3$ counts per second) becomes smaller. Note that all maxima are obtained for $\theta = 44°$ and $\phi = 0°$. We followed the error analysis in Sec. I.

[1] Razavi, M., Söllner, I., Bocquillon, E., Couteau, C., laamme, R. & Weils, G. Characterizing heralded single-photon sources with imperfect measurement devices. *J.*

*Phy. B* **42**, 114013 (2009).

[2] Bashkansky, Mark., Vurgaftman, Igor., Pipino, Andrew C. R., & Reintjes, J. Significance of heralding in spontaneous parametric down-conversion. *Phys. Rev. A* **90**, 053825 (2014).
